# Tracing the Invasion of the Mediterranean Land Snail *Cornu aspersum aspersum* Becoming an Agricultural and Garden Pest in Areas Recently Introduced

**DOI:** 10.1371/journal.pone.0049674

**Published:** 2012-12-05

**Authors:** Annie Guiller, Marie-Claire Martin, Céline Hiraux, Luc Madec

**Affiliations:** Centre Nationale de la Recherche Scientifique, UMR 6553, University of Rennes, Rennes, France; CNRS, Université de Bourgogne, France

## Abstract

This study is the first on the genetics of invasive populations of one of the most widely spread land mollusc species known in the world, the “Brown Snail” *Cornu aspersum aspersum*. Deliberately or accidentally imported, the species has become recently a notorious pest outside its native Mediterranean range. We compared the spatial structure and genetic variability of invasive (America, Oceania, South Africa) *versus* native populations using five microsatellite loci and mitochondrial (Cyt *b* and 16S rRNA) genes as a first step towards (i) the detection of potential source populations, and (ii) a better understanding of mechanisms governing evolutionary changes involved in the invasion process. Results based on multivariate analysis (Discriminant Analysis of Principal Components), Bayesian statistical inference (Clustering, Approximate Bayesian Computation*)* and demographic tests allowed a construction of the introduction pathways of the species over recent centuries. While emigrants originated from only one of the two native lineages, the West one, the most likely scenario involved several introduction events and “source switching” comprising (i) an early stage (around 1660) of simultaneous introductions from Europe (France, Spain) towards Oceania (New Zealand) and California, (ii) from the early 18^th^ century, a second colonization wave from bridgehead populations successfully established in California, (iii) genetic admixture in invasive areas where highly divergent populations came into contact as in New Zealand. Although these man-made pathways are consistent with historical data, introduction time estimates suggest that the two putative waves of invasion would have occurred long before the first field observations recorded, both in America and in Oceania. A prolonged lag period as the use of an incorrect generation time could explain such 100–150 years discrepancy. Lastly, the contrasting patterns of neutral genetic signal left in invasive populations are discussed in light of possible ways of facing novel environments (standing genetic variation *versus* new mutation).

## Introduction

Elucidation of the mechanisms driving the success of invasive species requires detailed knowledge of the phylogeography and genetic characteristics of both source and invading populations. A large number of studies devoted to invasive taxa have increasingly demonstrated the importance of identifying the geographic origin and lineage sources of introduced populations and the mode and pathways of their introduction in order to determine factors influencing successful invasions, to design management strategies and to prevent further spread of species which sometimes turn into agricultural pests [1]. While phylogeographical investigation is essential to detect potential native clades which could provide invaders pre-adapted for invasiveness [2], [3], [4], formal population genetics models might explain the geographical distribution of neutral genetic diversity within invasive *versus* native populations to understand and predict biological invasions. However, to avoid attributing present genetic changes to post-invasion genetic or ecological events, whilst they must simply reflect genetic differences in source populations, the original variability might be assessed with as much precision as possible [5], [6]. Such calibration is all the more difficult if the genetic diversity is high in the source area [6], [7]. To overcome the time constraint, efforts should also be made to standardize the sampling of introduced populations since major genetic changes generally occur during and after the introduction process.

Amongst the countless examples of invasive species that have been accidentally or intentionally introduced during the 19^th^ century, the Mediterranean mollusc *Cornu aspersum* is one of the most widely spread land snails in the world [8]. This polytypic species is divided into two subspecies, the farm reared ‘gros-gris’ or *C. a. maximum* whose origin is still doubtful since its range has not been investigated, and the common form ‘petit-gris’ or *C. a. aspersum*. Most probably native to north Africa [9], [10], the *aspersum* subspecies shows strong discontinuities between two well-defined geographical groups indicative of independently evolving lineages [10], [11], [12], [13], [14], [15], [16], [17]. The combination of spatial patterns inferred from variation of shell, genital and molecular characters in more than a hundred populations representative of the *aspersum* form (western Mediterranean and European coastlines) strongly indicates a deep genetic split into an Eastern and a Western lineage. The East one (lineage C) includes especially samples from East north Africa (from East Kabylia to Tunisia), and the West one (lineage B) consists of populations from western north Africa (from western Kabylia to Morocco) and Europe. Signatures left in the geographical distribution of the genetic variation suggest that Pliocene and Pleistocene events involving vicariant and dispersal processes through tectonic changes and climatic fluctuations would explain such an East-West genetic split, also described in other co-distributed taxa (see [10] for details). Support for this phylogeographical pattern in the native range of the species is shown by (i) a limited dispersal of the typical eastern lineage after the last glacial maximum (about 18 000 years BP), (ii) the differentiation of the western lineage from eastern ancestral types, (iii) a northward colonization from north Africa to Europe of the western lineage via Tyrrhenian/Aegean routes or the Strait of Gibraltar [10].

The typical anthropogenic *aspersum* form is now present in many zones having Mediterranean, temperate and even subtropical climates, on the American and African continents, as well as on the Mascarene Islands, Oceania and Asia [8]. More recent events related to human activities are more likely responsible for long distance dispersal. As the ancient Romans were very fond of snails, *C. a. aspersum* may well have been introduced early on by these invaders who would be the first people rearing snails [18]. Besides intentional introductions for farming purposes to produce food, it has been accidentally introduced by the movement of plants and vegetables. Quarantine prevention measures for plant material have been adopted in several states in the USA and in Canada in the 1970s.

The post-Pleistocene geographical spread of the brown garden snail is documented in several parts of the world. However, records are based on observational data that can sometimes be misleading regarding the number of introduction events in the same territory or the lag time between the first introduction and the first field observation. For example in North America, the species would have been introduced into California during the 1850s for use as food by French immigrants [19], [20]. By 1900, *C. a. aspersum* was present throughout much of the agricultural area of California and it has been regarded as a pest in citrus orchards since that time [21]. In the Pacific, the oldest introduction recorded was in New Caledonia in 1879 [22]. Very common in New Zealand, it has also become a pest there. There is no precise information on the mode and date of introduction except that the species was deliberately imported from Europe for food [23], [24]. The species is also present in the Hawaiian Islands, where it was first recorded in O’ahu in 1952, then in Kaua’i in 1965 and 1976, in Hawai’i in 1976 and in Maui in 1981 [25]. Dubious records also exist for the non-native zones of Africa. The species has been introduced in South Africa as a food animal in 1855 with a first observation recorded in Cape Town (DG Herbert, Natal Museum, South Africa, pers. comm.). However, Herbert suggests that *C*. *a. aspersum* was probably introduced accidentally long before this, perhaps since 1650–1700.

Whilst our biological model suffers from scarce or speculative introduction records in invasive regions, it benefits greatly from intensive studies on genetic diversity and phylogeography in its native range [10], [12], [13], [14]. The introduction history of this species on which depends the genetic diversity and the evolutionary potential of introduced populations could therefore be partly elucidated by exploring biogeographical patterns as previously suggested. A wide geographic coverage of populations uniformly sampled throughout the entire native range has the potential to identify the original sources of introductions.

The aim of this study, devoted for the first time to invasive populations of *C. a. aspersum*, is to answer the following questions arising from the spatial structuring of the genetic variability observed in its native and invasive ranges. (i) If introduced populations originated from one or two lineages? and if both lineages were involved, did the East and West lineage supply roughly equal numbers of migrants? (ii) Whatever the source lineage, which introduction scenario can be inferred from the genetic signature left in introduced populations? Did migrants originate from a single or multiple introduction events? In the case of multiple introductions, which are often thought to account for the invasive success of genetically variable populations [26], [27], [28], are they attributable to one source of introductions repeated several times, or to several distinct sources? Of the introduced populations which became established, could some have acted as a founding for further long distance dispersal of migrants into new areas? This spread by stages or “source switching” to avoid confusion with the stage-based model used to characterize the invasion process [29], is called the invasive bridgehead effect [30], [31]. This specific modality of introduction, inferred from the invasion scenarios of several taxa is more than a simple and descriptive pathway of initial introduction [26], [30], [32], [33], [34], [35]. Compared to multiple introductions which occur independently from native populations, such a spreading process would be more advantageous since evolutionary changes required to disperse and successfully establish would arise only once, i.e. in the bridgehead population. So, do such founding populations providing second generation invaders, exist in our biological model? The overrepresentation of the West lineage in Europe compared to the uncommon and scattered East-type populations suggests that discrepancies in the success of northward expansion would reflect variation in adaptive potential of populations among West and East lineages. Although local selective pressures can differ greatly in newly invaded environments, one can expect for the wide range of ecological conditions of native habitats, that the successfully established populations are exclusively native to the West lineage. Whilst individuals of the East lineage might attempt invasions, it seems that they did not survive and introduction failed.

## Materials and Methods

### Samples

**Figure 1:**
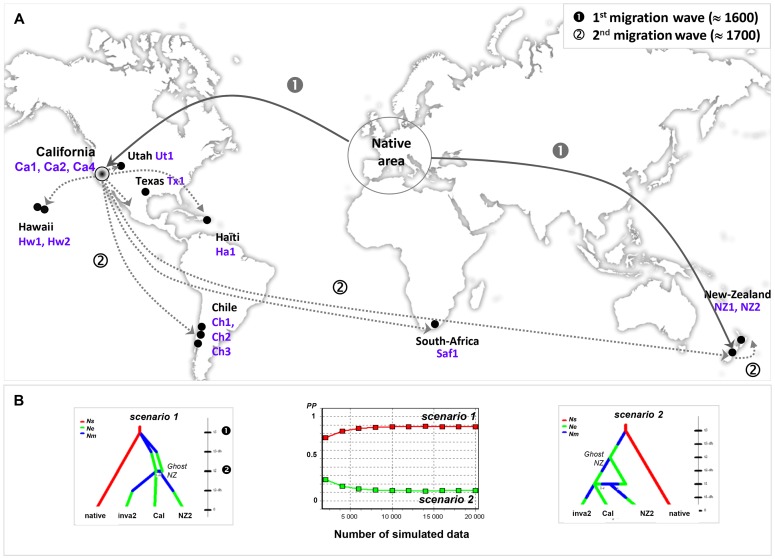
Worldwide native and invasive ranges of *C. a. aspersum* and likely scenario of invasion. A: Sampling locations of the 14 invasive populations analyzed and introduction pathways (routes and periods) from the Mediterranean native area inferred from Diy ABC analysis (population codes and numbers are given in the Materials and Method section and in Table S1). B: Schematic representation of the two hypothetical scenarios tested using Diy ABC to infer population introduction especially in New Zealand (NZ_2_), and graph of logistic regression showing the posterior probabilities of both scenarios tested (see Materials and Methods section for description of scenario 1 and 2).

Specimens of the native area were either previously investigated (north Africa, continental Spain, Portugal, continental Italia, Sardinia, continental Greece, Crete, Croatia, Turkey, continental France, Corsica, UK; [10], [13] or newly analyzed (Corsica, Malta, Sicily, Balearic Islands). Samples from invasive populations were obtained through extensive collaborations and were collected from 14 localities: North America (3 samples from California, Ca_1_, Ca_2_, Ca_4_; 1 sample from Texas, Tx_1_; 1 sample from Utah, Ut_1_), Hawaï (2 samples - Maui, Hw_1_; Big Island, Hw_2_), South America (Chile, 3 samples Ch_1_, Ch_2_, Ch_3_), Africa (1 sample from South Africa, SAf_1_) and Oceania (2 samples from New Zealand, NZ_1_, NZ_2_) (Figure 1a). All specimens were collected recently (from 2005 to 2011) except those from Texas (June 1975) and Utah (August 1983) sent by Jochen Gerber (specimens donated by J Gerber, Collections Manager at Field Museum of Natural History, Division of Invertebrates, Chicago, USA). In all, 266 invasive snails were examined. Individuals of the subspecies *maximum* coming from a snail farm in Brittany (France) were also analyzed and used as an outgroup in phylogenetic and network-based analyses. Sample site characteristics and haplotype codes are given in Table S1. Both native and introduced populations were freely collected since the sampling of *C. a. aspersum* individuals required no relevant permission.

Since the computation of probabilities is very time consuming for large data sets, phylogenetic analyses based on mitochondrial sequence variation (cyt *b versus* 16S) consisted of two steps. First we performed a preliminary analysis using all native individuals (390 and 405 sequences for 16S and cyt *b* genes respectively) and then, we discarded groups of populations or lineages that could not be the source of the invasive individuals. The resulting reduced data set comprised 262 sequences for cyt *b* (192 native and 70 invasive) and 263 sequences for 16S (188 native an 75 invasive). For nuclear variation, 587 individuals were genotyped for polymorphism in five microsatellite loci but only 424 (252 native and 172 invasive) were used after removing individuals with missing data (Table S2).

### DNA Extraction, Amplification and Sequencing

Total genomic DNA was obtained from foot and mantle muscles of fresh or alcohol-preserved material using the chelex extraction protocol [13]. For phylogeographical analyses, we amplified a segment of approximately 380 and 560 bp for the 16S rRNA and cyt *b* mitochondrial genes respectively (see [10] and [36] for details). For population genetics, only five out of 17 microsatellite loci previously identified [37] were examined and multiplexed in two distinct PCR reactions (Ha2, Ha10 and Ha11; Ha6 and Ha8). All other loci showing numerous null alleles could be effectively discarded. Locus amplification and allele identification were performed as reported in [37] with slight modifications.

### Mitochondrial Data Analysis

Mitochondrial sequences were aligned using the built-in assembly algorithm of the CodonCode Aligner software (v3.5, CodonCode Corporation, Dedham, Massachusetts). For the 16S region, they were manually adjusted, based on the secondary structure of the large ribosomal subunit of *C. nemoralis* [38]. New sequences produced were submitted to GenBank (Table S1). Analyses of sequence polymorphism were carried out with DnaSP v4.10.9 [39] and Arlequin v3.1 [40]. To infer phylogenetic relationships among individuals, we performed maximum likelihood (ML) and Bayesian-based inference (BI) methods as described in [10] by using Phyml v2.4.4 [41] and MrBayes v3.1.1-p1 [42] for ML and BI analyses respectively. For both genes, the best fit model of nucleotide substitutions tested with MrAIC v1.4.2 [43] was HKY [44]. Relationships between haplotypes of both mtDNA regions were estimated using median joining (MJ) networks implemented in Network v4.2.0.1 [45].

### Nuclear Data Analysis

#### Genetic diversity of source versus invasive populations

Standard population parameters were estimated to describe genetic variability within and between samples. Allele number, allelic richness, allele frequencies, observed heterozygosities and unbiased estimates of expected heterozygosities under Hardy-Weinberg expectations were calculated for each population and locus using Genepop v4.0 [46]. The fits of the data to Hardy-Weinberg expectations and genotypic disequilibria were also analyzed with Genepop. Tests for difference among groups of native *versus* introduced populations for several statistics (allelic richness, observed heterozygosity, gene diversity and F_ST_) were carried out using comparisons among groups of samples as implemented in Fstat v2.9.3.2 [47] with 15 000 permutations of individuals among groups tested. We also performed a multivariate analysis of variance (MANOVA, Minitab version 15) with 10 native and 10 invasive populations to examine the effects of population origin (native *versus* invasive status, location nested in status) and locus on allele number (*Na*), allelic richness (*A*) and heterozygosity (observed *Ho* and expected *He*) estimates. Non-normal data were first transformed by applying the Box-Cox transformation.

#### Population structuring

To detect population genetic structure and explore the relationships between the introduced populations and possible source populations, we used multivariate (Discriminant Analysis of Principal Components, [48]) and Bayesian clustering analyses, both of which were based on multilocus genotypes. The DAPC was performed with Adegenet v1.2–3. [49], an R package dedicated to the multivariate analysis of genetic markers. We first performed DAPC with populations used as prior clusters and then with unknown prior clusters. In that case, the number of clusters (*k*) or homogeneous groups of genotypes was assessed using the K-means clustering approach. An increasing number of clusters (*k*) were tested and the optimal *k* value was chosen on the basis of the lowest associated Bayesian information criterion (BIC) provided for each *k* tested. Misclassified individuals or individuals not assigned in the population where they were sampled could then be potential migrants. In the same way, homogeneous groups clustering individuals originating from different populations could suggest multiple introductions.

Clustering of individuals was investigated using Bayesian algorithms implemented in Structure v2.3.3 [50] software. Two different models for the ancestry of individuals were used to assign individuals in populations close to Hardy-Weinberg equilibrium with complete linkage equilibrium between loci. Whereas both models assume recent ancestry of individuals (admixture) and current gene flow (correlated allele frequencies) between populations. they mainly differ in the source of information provided. The first model (model 1) uses only genotype data to assign individuals to *k* virtual populations. For each value of *k* (ranging from 1 to 20), we carried out 20 independent Markov Chain Monte Carlo (MCMC) runs with 7500 iterations discarded as burn-in followed by an additional 20 000 generations. The optimal number of clusters *k* was verified by estimating the *ad hoc* statistic Δ*K* suggested by [51]. Once the true number of clusters was assessed, we generated 20 replicates using sampling location of populations as prior information (LOCPRIOR option) to assist clustering (model 2). All replicates of the *k* value inferred were aligned using Clumpp v1.1.2 software [52]. As *k* ≈ 15, we used the *LargeKGreedy* algorithm with *G*’ and 1000 random input orders. The Distruct v1.1 software [53] was used to graphically display the output from Clumpp.

### Inferring Alternative Invasive Scenarios Using ABC

The high level of genetic diversity and evidence for population admixture in one New Zealand population (NZ_2,_ see Results section) led us to test competing scenarios of the initial introduction of the species in that region. The relative likelihood of each scenario was estimated using the computer program Diy ABC [54] whose inferences are based on Approximate Bayesian Computation (ABC). Scenarios were customized to fit the first recorded introduction of the species during the middle of the 19^th^ century. Based on the geographic distribution of the mtDNA lineages and the genetic admixture at microsatellite loci found in NZ_2_, samples were pooled into one “native” and three introduced populations: “NZ_2_” for admixed invasive NZ_2_ population, “Cal” for invasive Californian populations, “inva2” for all other invasive populations. A fifth population considered as a ghost population (“ghost NZ”) was also included in both scenarios tested. This unsampled population acts as a parent in the admixture event occurring in New Zealand. The admixture of genetically diverse source populations in NZ_2_ could then be explained by (see also Figure 1b):

- scenario 1: two independent introductions occurred simultaneously from native range (“native”) to New Zealand (“ghost NZ”) and California (“Cal”) at time *t3*. At *t2*, Californian populations serve subsequently as source of invaders for newly invaded regions (“inva2”) and New Zealand gives birth at that time to the admixed population “NZ_2_”.
- scenario 2: New Zealand (“ghost NZ”) was first colonized by native migrants (“native”) at *t3* and then served as source of colonists for California (“Cal”) at *t2*. A second wave of introductions occurred from California to other regions (“inva2”) leading to admixed populations in New Zealand (“NZ_2_”) at *t1*.

We first simulated genetic data set under scenario 1 and then used this simulated data set as a test data set to compare both scenarios and evaluate the one that fits better the observed data. The effective population size is set to *Ne* = 5000 for “native” and *Ns* = 1000 for all introduced populations. However, new established populations started by small numbers of migrants (*Nm* = 25 diploid individuals per population) so that *Ns* was reached in introduced areas after a few generations (*db* = 5). Each individual was genotyped at five microsatellite loci and two mitochondrial genes. Microsatellite loci are assumed to follow the SMM mutation model, each locus has a mutation rate drawn from a Gamma distribution (mean = mean mutation rate and shape = 2). The two mtDNA genes are assumed to be of 600 and 400 bp and to follow the HKY model (% invariant sites = 40, α = 0.30). Approximate date of first introductions recorded in California were used to define the prior distribution of the coalescence time in all scenarios (*t1* = 75, *t2* = 100 and *t3* = 200). A number of 10^6^ data sets were simulated per scenario following the parameter values drawn from the prior distribution. To compare scenarios, we computed the posterior probability of each of them by performing a logistic regression based on 1% of the simulated data closest to observed data.

### Demographic Analyses

The dynamics of demographic expansion before and after introduction was inferred using tests of population growth. As described in [10], we used Fu’s *Fs* [55] and Ramos-Onsins and Rozas’ [56] *R*_2_ statistics which were estimated using DnaSP v4.10.9 [39] and Arlequin v3.1 [40] respectively. Evidence for population growth was also obtained from an examination of mismatch distributions [57], [58].

## Results

### Network Relationships between Native and Invasive Haplotypes

**Figure 2:**
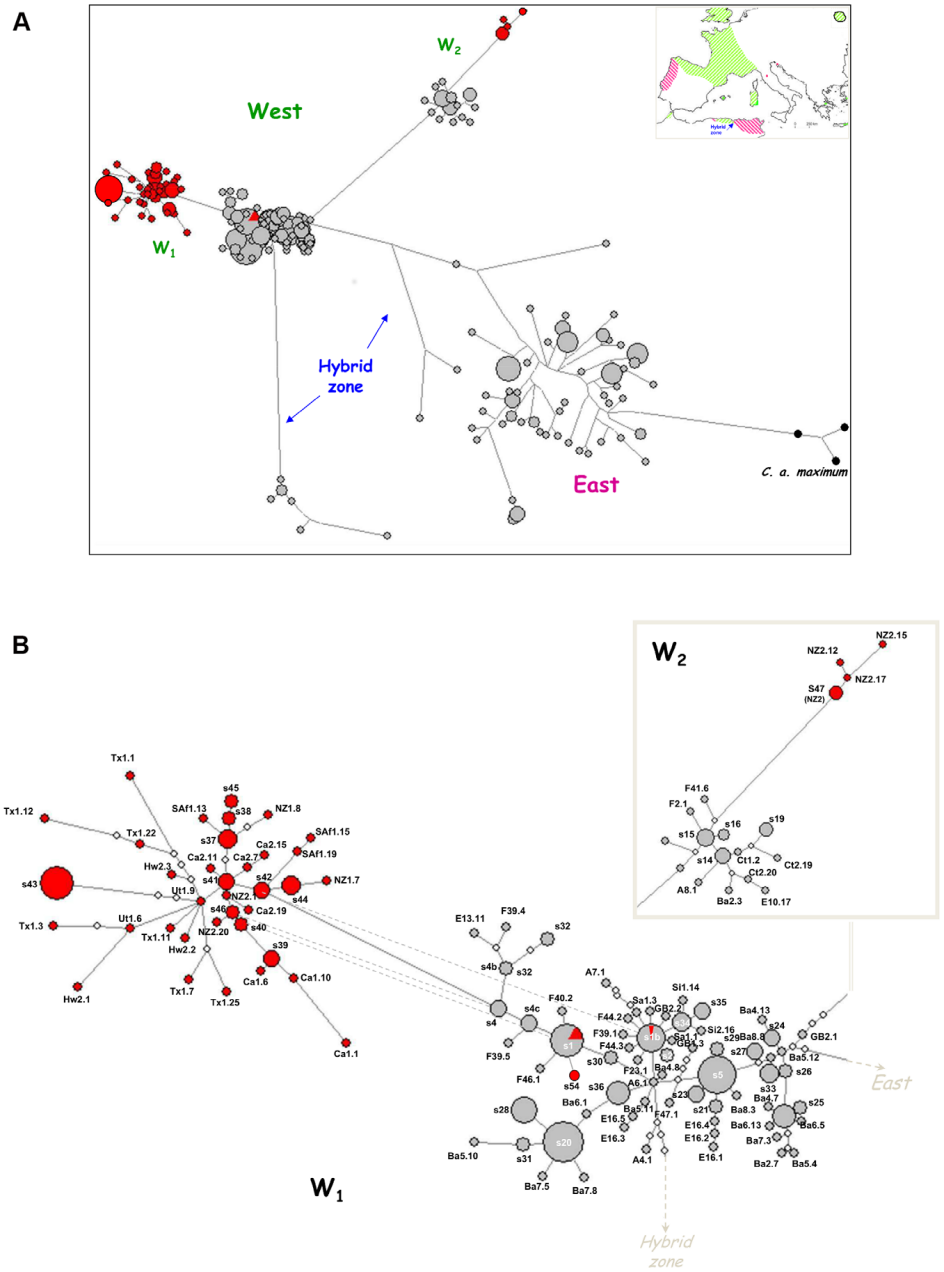
Median-joining network for the 16S rRNA mtDNA haplotypes of *C. a. aspersum*. Each circle represents a haplotype, and circle size is proportional to haplotype frequency. Colors indicate native (grey) *versus* invasive (red) status of haplotypes. Branch lengths are approximately equal to inferred mutational steps. A: Phylogenetic relationships of 390 individual sequences of *C. a. aspersum* and schematic geographic location of the main haplogroups defined (East *versus* West lineages are represented by pink and green color respectively, the Kabylia putative hybrid zone is in blue). *C. a. maximum* is used as outgroup. B: Focus on the relationships inside the West lineage represented by the W1 and W2 sub-lineages. Dashed lines represent superflous links deleted using the Network’s MP option. Haplotype codes according to those in Table S1.

**Figure 3:**
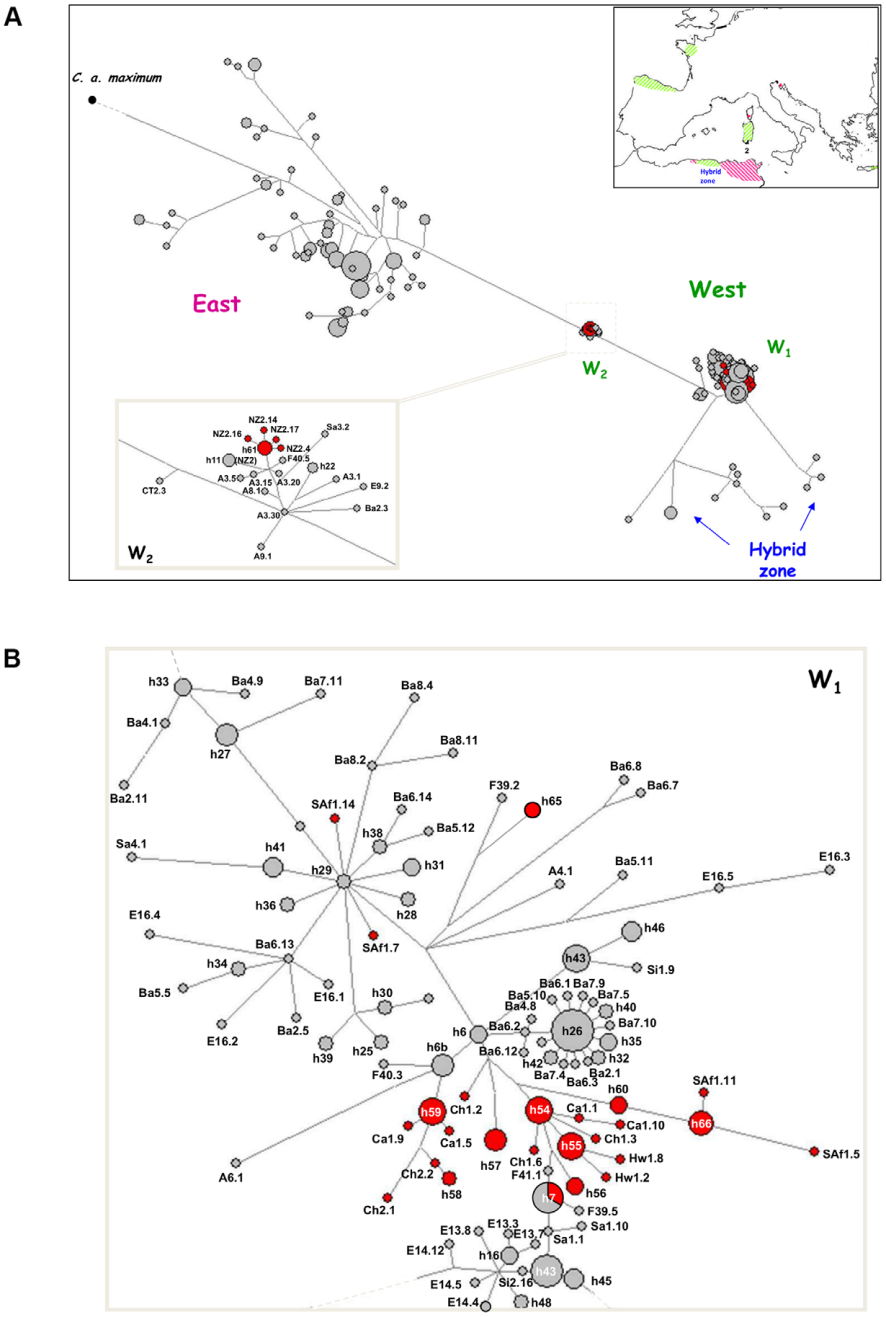
Median-joining network for the cyt *b* mtDNA haplotypes of *C. a. aspersum*. A: Phylogenetic relationships of 406 individual sequences of *C. a. aspersum.* B: Focus on the relationships inside the West lineage represented by the W1 and W2 sub-lineages (see legend of Figure 2 for details).

The first graph generated showed the clustering of all invasive sequences outside the East lineage (figure not shown). Subsequent analyses were made with most (140 and 126 haplotypes for cyt *b* and 16S respectively) eastern haplotypes excluded from data sets. For both genes, all invasive haplotypes linked to the West lineage were scattered throughout the main sub-lineage W1 except some haplotypes exclusive to the New Zealand population NZ_2_ (Auckland, North Island) which were part of the haplogroup W2 (Figures 2 and 3). In NZ_2_, four and five haplotypes (out of seven and six identified for 16S and cyt *b* respectively) distributed in a star*-*like pattern belong to the sub-lineage W2. The putative source populations of NZ_2_ haplotypes varied between the two sub-lineages but also between genes; 16S and cyt *b* individuals of W2 could only have originated from native-range source populations (Algiers area and Europe) while individuals of W1 would have diverged after secondary introductions from previous ones that occurred earlier in America or closer geographically in the South Island of the country. All remaining exotic 16S haplotypes of the W1 haplogroup were specific to introduced populations and diverged greatly from native ones except s54 arranged around the widely distributed common haplotype s1 shared by Californian (Ca_4_), Chile, French and Sicilian individuals (Figure 2b). The common haplotype s1b was also shared by native (French, Spanish) and invasive (Californian) populations. From the internal/external position of introduced haplotypes, Californian ones appeared to be older than those found in other North American samples (Texas, Utah and Hawaii), Chile, South Africa and the South Island of New Zealand. For cyt *b*, weaker divergence was observed between native and invasive haplotypes of W1, and apart from h7 found in France, Sardinia and California (Ca_4_), they were all specific to invasive samples (Figure 3b). For the ancestors of North-American colonists, both genes indicated that they have probably originated from Europe (Spain and France).

### Nuclear and Mitochondrial Diversities in Native and Invasive Populations

Estimates of genetic diversity based on microsatellite loci are shown in Table 1. MANOVA tests revealed a significant multivariate effect on *Na*, *A* and *Hs* for the three factors tested, i.e. invasive *versus* native status (Wilks’ λ = 0.283, *F* = 62.5, p<0.001), locus (Wilks’ λ = 0.462, *F* = 5.53, p<0.001) and population (Wilks’ λ = 0.364, *F* = 1.66, p = 0.006). Thus, allelic richness (*A*) and gene diversity (*Hs*) were significantly lower in the invasive populations than in the native ones. However, univariate tests (ANOVA) showed that population effect was only significant for *Na*. Matrix of pairwise correlations showed a relative decrease of the partial *r* only between *He* and *Na* but it remained highly significant (*r* = 0.57; p<0.001). Results from mtDNA mirrored the lower nuclear diversity in invasive populations (Table 2). Nucleotide diversity values were effectively twice and 3.5× higher in native populations than in invasive ones for 16S and cyt *b* respectively. The within-population divergence in NZ_2_ was high compared to estimates of other invasive populations and the relatively low haplotype diversity found (Table 2).

**Table 1:** Genetic statistics comparing native and invasive populations for five microsatellite loci.

Range	N	*Na*	*A*		*Ho*	*Hs*		*Fst*
**native**	*10*	**11.22**	a **8.69**	***	**0.659**	**0.822**	**	**0.115**
		*3.82*	*2.61*		*0,143*	*0.070*		*0.014*
**invasive**	*10*	**7.60**	^b^ **5.05**	***	**0.652**	**0.745**	**	**0.152**
		*2.38*	*1.25*		*0.112*	*0.126*		*0.014*
**All pop.**	*20*	**9.41**	^b^ **5.54**		**0.638**	**0.775**		**0.137**
		*3.10*	*1.34*		*0.111*	*0.069*		*0.014*

N; number of populations; *Na*: mean number of alleles per locus; *A:* allelic richness; *Ho*: mean observed heterozygosity over all loci; *Hs*: mean gene diversity over all loci; *Fst*: theta (*F_ST_*) over all loci. Standard errors are indicated in italics and in brackets. Tests for difference among groups of populations were assessed using the procedure implemented in Fstat with 15 000 permutations of individuals among groups tested (*<0.05; **<0.01; ***<0.001).

a and ^b^are based on minimum sample size of 14 and 6 diploid individuals respectively.

**Table 2:** Genetic diversity estimates and results of demographic tests for cyt *b* and 16S genes in main geographical subdivisions of native and invasive *C.a. aspersum* range.

	**Range**	***N***	***h***	**Diversity index**		**Demographic tests**			
	**Populations**			***H*** ** ± sd**	***θπ*** ** ± sd**	***Fs***		***R*** **_2_**	
**Cyt ** ***b***									
	**Invasive**								
	SAf_1_	10	5	0.667±0.163	4.22±2.29	1.14		0.141	
	USA	28	12	0.881±0.041	4. 00±2.06	−2.12		**0.077**	**
	Ca_l_	20	10	0.905±0.041	4.57±2.35	−1.17		0.106	
	Hw_2_	8	3	0.464±0.200	1.00±0.75	0.20		0.250	
	Ch_1–3_	15	8	0.867±0.067	3.66±1.96	−1.09		0.103	
	NZ	17	8	0.882±0.047	27.40±12.63	7.85		0.231	
	NZ_1_	8	3	0.679±0.122	3.57±2.03	3.00		0.242	
	NZ_2_	9	6	0.833±0.127	12.19±6.09	2.05		0.281	
	**all**	70	30	0.951±0.011	14.42±6.54	−1.64		0.084	
	**invasive - NZ**_2_	61	24	0.942±0.013	5.05±2.48	−**8.64**	**	**0.057**	*
	**Native**								
	**all**	332	190	0.990±0.002	52.18±22.62	−**23.70**	***	0.102	
	**West**	192	109	0.982±0.004	20.48±9.09	−**23.72**	***	0.054	
**16S**									
	**Invasive**								
	SAf**_1_**	9	5	0.806±0.120	3.94±2.18	0.66		0.178	
	USA	45	25	0.980±0.013	7.03±3.39	−**12.90**	***	**0.065**	***
	Ca**_l_**	22	14	0.952±0.026	5.61±2.80	−3.66	*	0.116	
	Hw**_1–2_**	12	4	0.455±0.170	5.73±2.95	4.18		0.130	
	Tx**_1_**	7	7	1.000±0.076	9.62±5.03	−1.63		**0.191**	***
	NZ	19	12	0.936±0.037	15.16±7.09	0.94		0.141	
	NZ**_1_**	10	6	0.844±0.103	5.47±2.87	0.60		0.157	
	NZ**_2_**	9	7	0.917±0.092	14.72±7.29	1.09		0.200	
	**all**	75	43	0.932±0.015	11.84±6.29	−**14.15**	**	0.098	
	**invasive -NZ_2_**	66	37	0.920±0.018	9.00±5.13	−**14.02**	**	**0.053**	*
	**Native**								
	**all**	314	159	0.987±0.002	20.56±9.11	−**23.55**	**	0.072	
	**West**	**188**	**85**	**0.973±0.005**	**8.60±3.99**	−**24.40**	***	**0.057**	

*West*: sequences of the West lineage only; *invasive-NZ2*: all invasive sequences excluding NZ_2_ ones; *N*: number of individuals sequences; *h*: number of haplotypes; *H*: gene diversity; *θ*π: nucleotide diversity; sd: standard deviation; *Fs:* Fu’s F statistic; *R*_2_: Ramos-Onsins and Rozas’ statistic; significance levels for *Fs* and *R*_2_:

* <0.05;

** <0.01;

*** <0.001.

### Population Structuring

**Figure 4:**
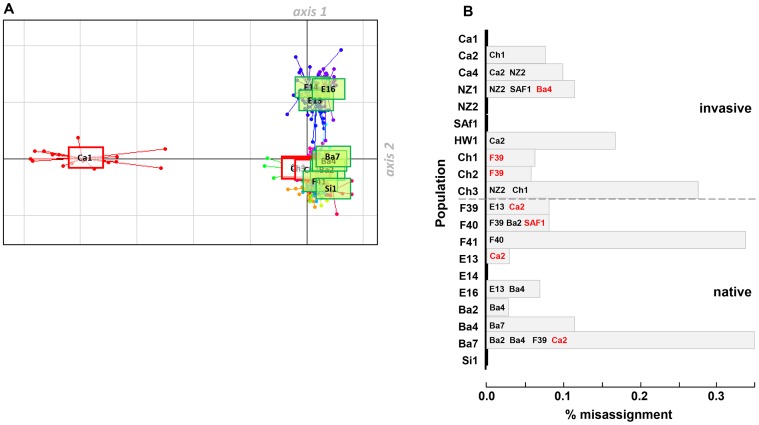
Results of the first DAPC performed with 10 native and 10 invasive populations as prior. A: Scatterplots of the first two principal components (first axis: 39.3% of total variance; second axis: 21.2% of the total variance) of the DAPC of 424 individuals genotyped at 5 microsatellite loci. Dots shown by different colours represent individuals of each population. Native and invasive populations are represented by green and red labels respectively. B: Percentage of miss-assignment per population with information on origin of native (black) and/or invasive (red) population(s) to which miss-assigned individuals would be clustered with the highest probability.

A first DAPC on genotypic data of 424 individuals (individuals with at least one missing genotype data were excluded) was made using all 20 populations as clusters (Figure 4a). The first two principal components clearly separated on the first axis for invasive individuals of one Californian sample (Ca_1_), whilst the second axis differentiated individuals from continental Spain (E_13_, E_14_ and E_16_). Such strong differentiation between invasive samples may explain the strongest F_ST_ value found for invasive populations (Table 1). The average percentage of individuals miss-assigned to the population where they were collected was low (8.5%), with 7.5% and 9.1% for invasive *versus* native sets of samples respectively (Figure 4b). Amongst miss-assigned individuals that could be possible migrants, three invasive individuals out of 13 were assigned to native populations (F_39_ and Ba_4_) and similarly, four native individuals out of 23 were assigned to invasive samples (Ca_2_, SAf_1_; Figure 4b). Regarding the highly diverse invasive NZ_2_ populations, no individual of this colony was miss-assigned, whilst three invasive individuals of NZ_1_, Ch_3_ and Ca_4_ would be classified in NZ_2_. A second DAPC was performed without prior sampling location information to assist the clustering of individuals. Amongst the *k* = 10 homogeneous groups of genotypes identified, the two first discriminant functions showed the differentiation of three groups (8, 1 and 9) clustering mainly native individuals from Spain and France since only two individuals from NZ_2_ and Ca_2_ joined groups 1 and 9 respectively. With a different “status-ratio”, all seven other groups defined clustered native and invasive individuals and were all weakly genetically differentiated from each other.

**Figure 5:**
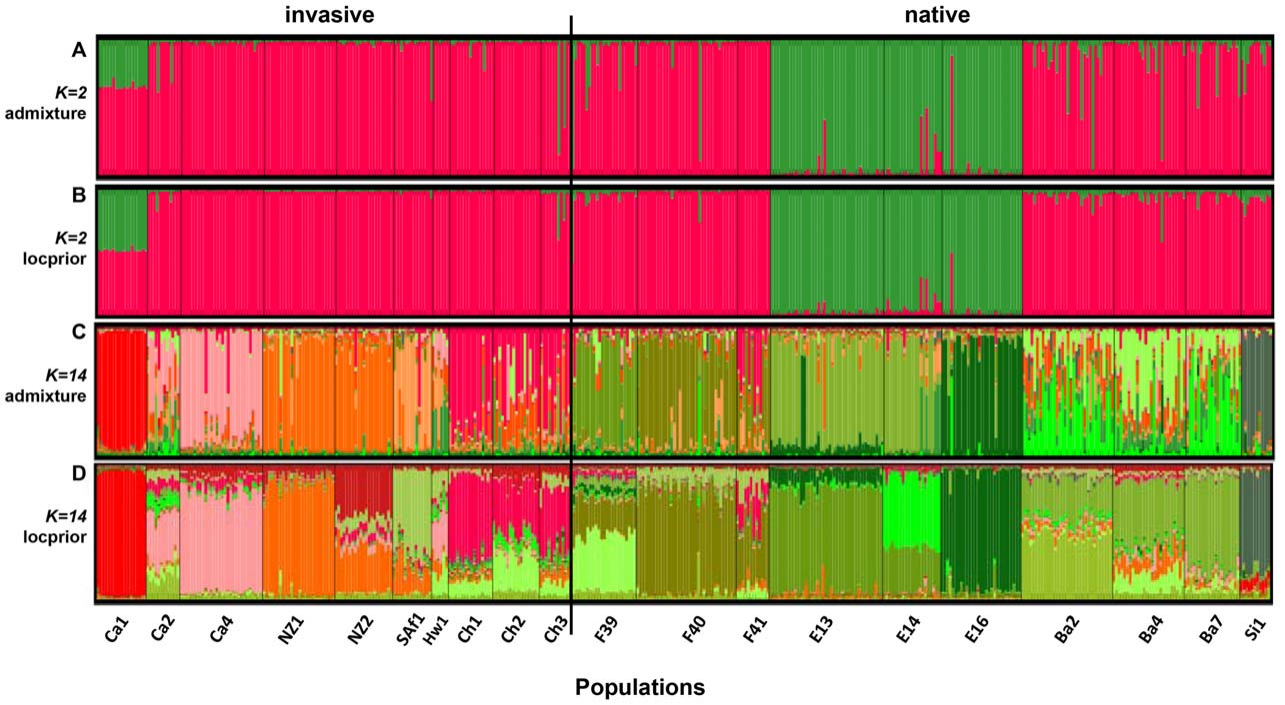
Population structure from Bayesian STRUCTURE analyses using 5 microsatellite loci for different values of *k.* Each distinct cluster is represented by a particular color. Each vertical bar represents an individual. A: Population clustering for *k = *2 and model 1 (admixture and allele frequencies correlated). B: Population clustering for *k* = 2 and model 2 (admixture, allele frequencies correlated, sampling location of populations as prior information). C: Idem as A with *k* = 14. D: Idem as B with *k* = 14 (see Materials and Methods section for models description).

Results of the Structure clustering indicated that the most parsimonious estimate of the number of clusters was *k* = 2. Whatever the model tested, the structure inferred mirrored results of the first DAPC with multilocus genotypes of the three Spanish samples assigned predominantly into one of the two clusters (Figures 5a, b) and genotypes from Ca_1_ showing an admixture pattern between the two distinct clusters. To describe more thoroughly the assignment of individuals other than Spanish ones, together with the bimodal peaks of Δ*k* indicating a level of substructure with 14 clusters, we examined the content of these clusters with and without prior sampling location information in the 14 groups inferred (Figures 5c, d). At *k = *14, Ca_1_ and Spanish individuals clustered separately. Ca_1_ appeared weakly admixed, meaning that in spite of common ancestors in Spain, individuals were sufficiently distinct to be segregated in a specific cluster. Whilst mean values of *r* (0.71±0.06) and α (0.53±0.10) suggested that sampling location was roughly informative and that each individual would have originated mostly from a single population, the LOCPRIOR model ignored the sample group information to cluster NZ_2_ individuals since NZ_2_ genotypes split into two main distinct clusters, with most individuals having ancestries both in NZ_2_ and NZ_1_. The ancestry of individuals seemed also uncorrelated with the sampling location of Chilean populations with fractions of their genome having ancestries in the French F_39_ (green) and F_41_ (red) populations.

### Inferring Migration Pathways of *C. a. aspersum* in Introduced Regions Using ABC

Posterior probabilities of scenarios estimated through a logistic regression showed better support for scenario 1 than for scenario 2 (Figure 1b), meaning that two independent introduction events would have occurred successively in New Zealand. Secondary contact between snails originating from two different waves of invaders, first from Europe and then from California, would then be responsible for the high level of within-diversity in NZ_2_. Contemporary invasions would occur from California to other places (“inva2”). The medians of the posterior distribution of *t3* and *t2* were 221 (95% CI = 83–296) and 156 (95% CI = 59–197) generations respectively. Assuming a generation time of two years, estimates (especially *t3* ≈ 442 years and *t2* ≈ 312 years) implied events which would have occurred over times much longer than the first introductions recorded around 1850 in California.

### Demographic Analyses

**Figure 6:**
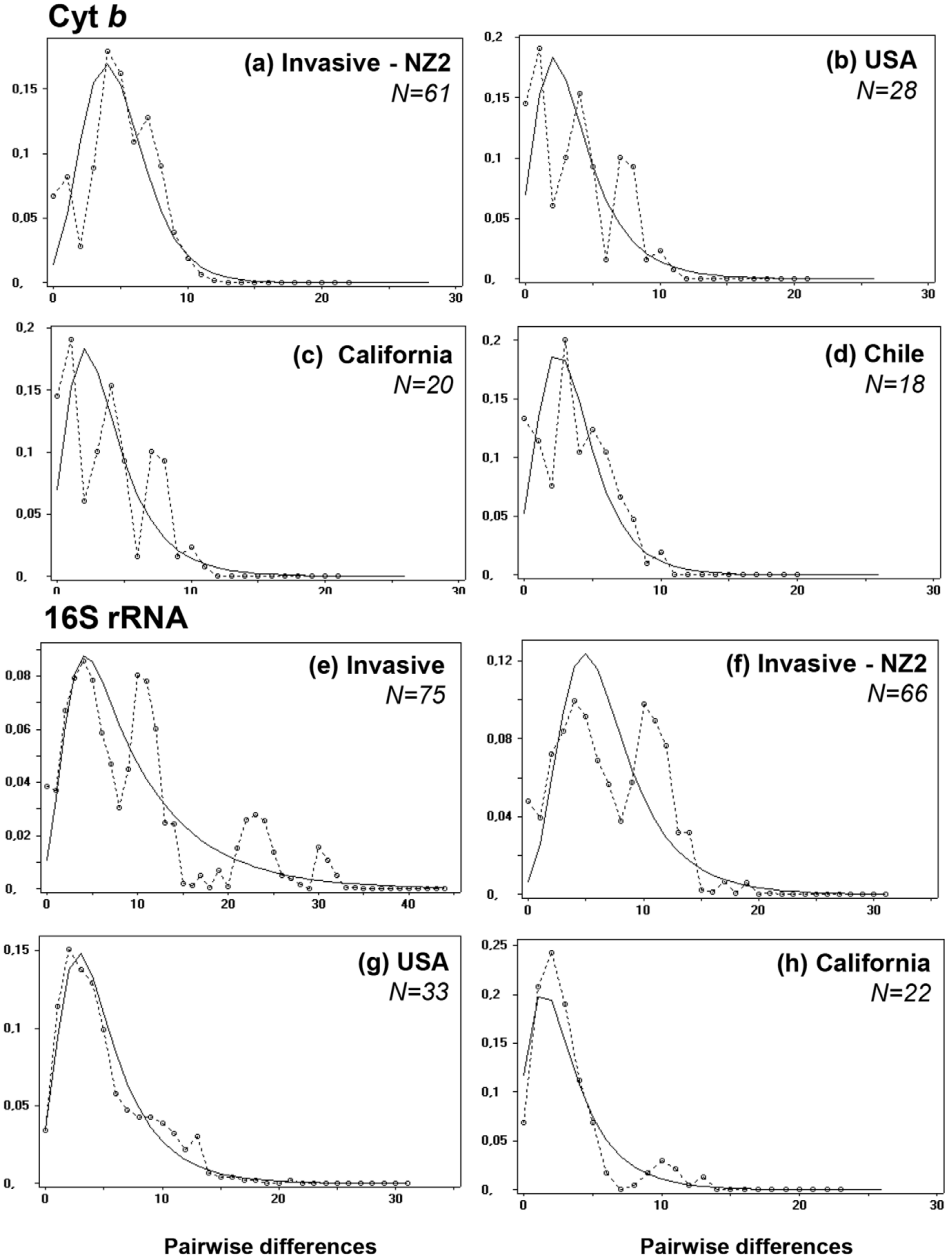
Mismatch distributions in native *versus* invasive subdivisions for cyt *b* sequences and 16S rRNA sequences. The continuous and interrupted (connecting circles) lines indicate the expected and observed distributions of pairwise differences obtained by fitting a model of sudden population expansion. Cyt *b* graphs based on (a) all invasive excluding NZ_2_, (b) North American, (c) Californian, (d) Chilean sequences. 16S rRNA graphs based on (e) all invasive, (f) all invasive excluding NZ_2_, (g) North American, (h) Californian sequences.

Estimates of demographic parameters for detecting population growth were performed in native *versus* non-native groups of populations (Table 2). Native samples included all available sequences of the native range (“native”) or only sequences relative to the West lineage (“West”). Regarding the strong genetic splitting observed between NZ_2_ and all other invasive populations, tests on invasive set of samples were performed with (“invasive”) and without NZ_2_ (“invasive-NZ_2_”). For the 16S gene, one or both Fu’s *Fs* test of selective neutrality and *R*_2_ statistics yielded significant results for “invasive”, “invasive-NZ_2_”, “native” and “native West” sets of populations. For cyt *b*, demographic expansion characterized “native”, “native West” and “invasive-NZ_2_^”^. Locally, tests performed on partial range of invasive populations indicated that the null hypothesis of constant size is not rejected for North-American populations either for cyt *b* as for 16S genes. These results were confirmed by the observed mismatch distributions which closely matched those expected under a model of a sudden expansion for these data sets (Figures 6a, b, e, f, g). Whilst Fu’s *Fs* and *R*_2_ statistics indicated that a model of constant size could not be rejected, unimodal distributions of pairwise differences among Californian haplotypes for both genes, and among Chile haplotypes for cyt *b* were also indicative of population expansion (Figures 6c, d, h).

## Discussion

### Unequal Contribution of Native Lineages in Providing Invaders

As predicted by the current distribution of mitochondrial lineages in native areas, variations at both nuclear and cytoplasmic gene loci unambiguously argue for an exclusive western origin of all introduced populations, with no detectable genetic admixture between western and eastern individuals. This geographic spread of the west lineage is moreover supported by the mtDNA signal of demographic expansion in western native populations. Besides evolutionary consideration implying higher competitive and reproductive performances of western typical individuals to account for their successful colonization in both native and invasive regions, the lack of eastern founders could result from the difference in probability sampling of both lineages. Indeed, members of East and West native populations do not have an equal chance of being selected since eastern populations are underrepresented compared to widespread western ones. However, for the eastern populations, the potential for evolutionary responses to novel environments would be limited in any case because of the small number of individuals that could be released at different times and places. As discussed below, the successful invasion of *C. a. aspersum* in some parts of the world seems effectively to depend on the number of introduction events. As for many species, multiple introductions especially in New Zealand would have been necessary to help overcome the severity of the genetic bottleneck induced by founder effects during introduction [26], [27], [59], [60].

### Genetic Diversity in Invasive Populations: Report and Role

Many theoretical and empirical studies have focused on how genetic diversity is maintained during the invasion process and how it affects the success of colonization. However, contradictory results prevent us from predicting genetic profiles, and hypotheses advanced to explain specific response cannot be applied to all the invasive populations or species concerned. Numerous studies have indicated that the fate of introductions is frequently determined by the propagule pressure [61], [62]. Many biological models showed a loss of allelic variation and expected heterozygosity in non-native populations [6], [63]. However, an increase in within-population diversity can be observed in the case of multiple genotypes released through a large number of individuals introduced at a given time or through repeated introduction events. Regarding successful invasive populations of *C. a. aspersum*, most of them show reduced allele richness and gene diversity compared to native counterparts. The NZ_2_ population stands out as an exception since its level of nucleotide diversity is higher than the value estimated from all invasive populations and, in particular, is higher when compared with native populations of the west lineage for the 16S gene especially. While not knowing the exact cause or role of genetic diversity, all invasive populations of *C. a. aspersum* should leave the same genetic signature if an increase of genetic variability was simply a side effect of the invasion process. Beyond phylogeographical pattern across New Zealand suggesting that admixture between divergent source populations would likely be responsible for surprisingly high levels of genetic variation in NZ_2_, such admixture does not seem to be essential for successful establishment in view of the loss of genetic variation in all other invasive populations. Unlike many other species for which multiple introductions provide most of the adaptive potential through the accumulation of disparate exotic variants and the creation of novel genotypes [26], [64], [65], such genetic mixing should be not necessary for *C. a. aspersum*. One argument in favor of this hypothesis is the extremely high level of genetic diversity found in source populations whatever the nuclear or mitochondrial markers analyzed [10], [11], [13]. Evidence supporting this prediction comes from cross-referring data from the different invasive populations of *C. a. aspersum* and from other invasive species showing that the genetic basis of invasiveness could all be found in standing genetic variation [66], [67], [68]. A better understanding of mechanisms governing evolutionary changes from "stored" variation will however require further investigation comparing the molecular signature of selection in introduced and native populations. As for many other invasive species, rapid adaptation to novel conditions could be mostly driven by changes in life history traits related to dispersal behavior [69]. In helicid snails, energetic cost of locomotor activity is one of the highest among animals, is phenotype-dependent and linked to the individual size and reproductive status [70], [71]. Thus, independently of human-mediated dispersal in the introduction and in the spread of the species, individual variation in dispersal would have an important impact on the colonization success of *C.a. aspersum*.

Overall, the fluctuating pattern of genetic variability in invasive populations of *C. a. aspersum* is another example demonstrating that the genetic paradox is a thing of the past [72], [73]. This raises the questions of the value placed on genetic diversity estimates through neutral molecular markers. Recent theoretical research and many case studies have effectively suggested that such estimates seem to poorly predict species’ invasiveness [6], [73]. The ability to invade does not appear to be very dependent on genetic variation of introduced populations meaning that a high level of genetic diversity does not seem to be a prerequisite for successful invasions. Genetic characteristics reported recently in the literature across different invasive species suggest that neutral variation does not correctly reflect the genetic diversity relevant to and required for invasive success [6], [73]. One argument supporting this observation is that quantitative traits would be less sensitive than neutral genes to a demographic bottleneck. The reasons are the loss of variation which is slower for polygenic traits than for individual loci and the stability of genetic variance since phenotypic variance would not be affected by the loss of rare alleles in bottlenecked populations. On the other hand, the negative consequences of reduced population size would be rapidly compensated for by an increase in genetic variance resulting from the conversion of non-additive to additive genetic variance [74], [75]. Although this slight effect is rarely observed in natural populations [76] or results in no significant change in adaptive response, such a transfer of variance components would explain higher levels of additive genetic variation in body size and tibia length in island populations of the moor frog *Rana arvalis* that experienced a recent bottleneck [77], or in desiccation resistance of the rain forest-restricted *Drosophila bunnanda* [78].

### Route of Invasion: a Putative Scenario

Although all founding snails originated from the western lineage only, both mtDNA and microsatellite markers indicate that highly distinct sources of western populations would have supplied migrants. The two evolutionary sub-lineages W1 and W2 identified in the native range participated in the colonization of North and South America, South Africa and Oceania although with unequal contributions. The haplotype distribution of cyt *b* and 16S genes indicates that 12 out of 13 invasive populations originated from the W1 sub-lineage. The spatial arrangement of the few shared haplotypes between native and invasive populations and their relatedness suggested, (i) a French or a Spanish origin of founders, (ii) an immigration pattern in several steps involving bridgehead populations located in California. The New Zealand population of Auckland (NZ_2_) is differentiated from all other invasive samples since some snails of this population fall into the distinct W2 sub-lineage without sharing haplotypes with native populations. As derived haplotypes of French and Spanish populations found in W2, the position of specific haplotypes of NZ_2_ would suggest that invaders for that New Zealand sample would have partially originated from Western Europe that has been originally colonized by migrants from the Algiers area in north Africa. Although they were described only in vague terms, historical data reinforce this assumption with a likely introduction of the species from Europe for producing food [19], [24]. Higher resolution of the spatial pattern of diversity based on subsequent sampling in both island and mainland parts of New Zealand will be able to indicate the exact place of first introduction and investigate the local factors that may have facilitated the invasion success.

Results of the Diy ABC analysis suggest a chronology of colonization events which occurred in New Zealand and indicate the direction of colonization. Since about 1600, two independent pools of immigrants would have dispersed simultaneously from native range towards New Zealand and California. More than one century later (around 1770), there is evidence for a second wave of invasion from successful populations established in California and introduced at varying distances throughout the world. The high level of genetic diversity observed in the population located in the North Island of New Zealand (NZ_2_) would then result from genetic admixture between highly divergent source populations, the ghost one (first invasion) supplanted by the arrival of Californian snails (second invasion). Haplotype relationships would suggest that the South Island (NZ_1_) first received Californian founders that subsequently expanded until they came into contact with snails of the first wave of introduction located in the North Island. Strong nuclear and mitochondrial affinities between South African and NZ_1_snails indicate that exchanges between the two continents could also have existed before and/or after the colonization of New Zealand by Californian invaders. Although the geographical situation of South Africa suggests that all scenarios are possible, the alternate derived or ancestral position of NZ_1_ and South African haplotypes both diverging from Californian ones seem to be unclear. Since accidental introduction of the species from the 17^th^ century would prevail over the intentional one recorded in 1855 (Herbert, pers. comm.), a colonization event from South Africa prior to the arrival of Californian snails cannot be ignored. Further analyses involving novel New Zealand and South African samples need to be conducted to shed light on exact source populations and routes of introduction of the species in Oceania. This would indicate whether early explorers used South Africa as a “port of call”. By contrast, the intermediate position of Californian snails specifically from Monterrey Bay (Ca_2_) provides evidence for the existence of a bridgehead effect [30]. Under this scenario supported by a clear signal of demographic expansion in California (Table 2; Figures 6c, h), the Ca_2_ population may have served as the source of colonists for secondarily invaded territories either closely located as other American states (Texas, Utah, Hawaii), or more distant geographically as Haiti, South America, South Africa and New Zealand. The notorious spread of this pest in North America and the earliest record of the species (around 1950) in the Hawaiian Islands support this bridgehead scenario [25]. The unequivocal deep nuclear differentiation of the Californian sample of Los Olivos (Ca_1_) also diverging from Ca_2_ reflects a strong genetic bottleneck in the founding population. The higher frequency of new alleles or alleles rarely found in other samples suggests that this population probably experienced a rapid recovery preventing the loss of genetic diversity while promoting rapid local adaption of invaders.

Although invasion pathways of *C. a. aspersum* in non-native areas are consistent with historical data, time estimates for introduction suggest that the two putative waves of invasion would have really occurred long before the first field observations were recorded, both in America and in Oceania. Two reasons might help to explain such a 100–150-year discrepancy. First, differences between estimates and historical records could correspond to the lag period between initial colonization and subsequent population expansion [79], [80], [81], [82]. Moreover this recovery period should be longer in the case of new mutations as the source for adaptive substitutions, as for example in the Californian population (Ca_1_). Unlike those drawn directly on standing genetic variation, novel alleles are indeed not immediately available [66], [83]. Second, the use of a wrong generation time is another possible cause of overestimated introduction dates. The average generation time of two years for *C. a. aspersum* could be reduced in introduced populations compared to native ones from which this time has been inferred. Due to a reduced density in pioneer populations, the rapid adaptation of some invasive species to face novel environmental conditions has been partially attributed to their capacity to change some of their life-history traits [84], [85], [86], [87], [88]. Such an increase in reproductive investment involving earlier maturity at a bigger size and a shorter generation time could thus explain the invasive success of the brown snail. Further work is however needed to relate the effect of population density on reproductive allocation and individual growth rate in invading populations of *C. a. aspersum*.

### Conclusion

This first study on the invasive populations of *C. a. aspersum* provides preliminary evidence for reconstructing the introduction pathways of the species over recent centuries. Not surprisingly, only immigrants of the western lineage would have been successful at all stages of the invasion process. However, the single origin of invasive lineage does not imply a simple colonization pathway. Strong genetic divergence among and within introduced populations suggests a scenario comprising (i) an early stage of two simultaneous eastward and westward introductions, (ii) a second colonization wave from bridgehead populations successfully established in California, leading to genetic admixture in invasive areas where divergent populations came into contact, such as in New Zealand. The contrasting patterns of neutral genetic signal left in introduced populations mirrored two different ways of managing novel environments, through (i) a high level of genetic diversity pre-existing in native populations and retained in bridgehead ones, (ii) the emergence of genetic novelties resulting either from new mutations or from admixture of divergent source populations. Although the relationship between genetic diversity and invasiveness seems to be not straightforward [73], previous results based on laboratory experiments suggest that such genetic potential would be associated with a considerable variation in life-history traits [89]. Further studies combining laboratory and reciprocal transplant experiments need to be conducted to link the variation of neutral genetic loci and phenotypic plasticity and to evaluate the adaptive value of variation in plasticity of traits underlying invasiveness. Whatever the nature of responses developed to face new environmental pressures, our results help to shed light upon evolutionary mechanisms that may favor the rapid and continue expansion of *C. a. aspersum* into a large range of habitats.

## Supporting Information

Table S1 Sampling characteristics (status, origin, geographic coordinates, population code, collectors, sampling date) of *Cornu aspersum aspersum* populations analyzed in this study and in [10], [13]. Population code: (i) Native: A, Algeria; Ba, Balearic islands; C, Corsica; Can, Canary islands, Ct, Crete; Cr, Croatia; E, Spain; F, France mainland; G, Greece mainland; GB, Great-Britain; I, Italia; M, Morocco; Mal, Malta; P, Portugal; Sa, Sardinia; Si, Sicily; T, Tunisia; Tu, Turkey; (ii): Invasive: Ca, California; Ch, Chile; Ha, Haiti; Hw, Hawaiian islands; NZ, New Zealand; SAf, South Africa; Tx, Texas; Ut, Utah). Mitochondrial diversity overall samples analyzed. Sample size (*n*), name of individuals sequenced (population code and individual number) and their corresponding cyt *b* and 16S RNA haplotypes and GenBank accession numbers. Two kinds of haplotype codes are provided: (i) the sequence name for unique haplotype, (e.g. A1.1), (ii) *hk* (*k* up to 71 distinct types) and *sk* (*k* up to 56 distinct types) for cyt *b* and 16S respectively for haplotypes shared by distinct individuals (*: haplotypes published in [13]; # haplotypes published in [10]).
(XLSX)

Table S2 Diversity measures from microsatellite loci comparing 10 native and 10 invasive populations of *C. a. aspersum*. *He*, expected heterozygosity per locus and per population; *Na*, number of alleles per locus and per population, *Ae*: allelic richness per locus and per population (based on minimum sample size of 6 diploid individual). Population code: (i) Native: Ba, Balearic islands; E, Spain; F, France mainland, Si, Sicily; (ii): Invasive: Ca, California; Ch, Chile; Hw, Hawaiian islands; NZ, New Zealand; SAf, South Africa. *n*: number of individuals analyzed per population; n*, number of individuals used for analyses after discarding individuals with too much missing data.
(XLSX)
